# How Can a Novel Molecular Diagnostic Assay Instill Confidence in Researchers and Encourage Its Future Use?

**DOI:** 10.5812/hepatmon.861

**Published:** 2012-04-30

**Authors:** Samad Amini-Bavil-Olyaee, Mahmoudreza Pourkarim

**Affiliations:** 1 Biotechnology Department, Pasture Institute of Iran, Pasteur Square, Pasteur Street, Tehran, IR Iran; 2 Laboratory of Clinical Virology, Rega Institute for Medical Research, Catholic University of Leuven, Leuven, Belgium; 3 Blood Transfusion Research Center, High Institute for Research and Education in Transfusion Medicine, Tehran, IR Iran

**Keywords:** Molecular Diagnostic Techniques, Hepatitis B Virus, Genotype

Dear Editor,

Determining hepatitis B virus (HBV) genotypes in a clinical setting not only aids therapeutic strategies but also expands the current knowledge of HBV molecular epidemiology. Recently many molecular approaches have been developed to differentiate different worldwide HBV genotypes, each approach with its own pros and cons. To establish such a critical method, several important concerns should be regarded [1][2]. Therefore, prior to releasing a molecular diagnostic approach and suggesting its usage for large-scale clinical and epidemiological studies, it is necessary to determine and verify the accuracy of the assay. We read with great interest the article written by Farazmandfar et al. regarding distinguishing HBV genotypes using type-specific primers, which is published by the “Journal of Virological Methods” [3]. The advantage of the method is that it can detect all eight HBV genotypes (A-H) in comparison to the similar method that was introduced before and was unable to detect HBV genotypes H and F [4]. To report a novel assay, all procedures should be defined precisely for other researchers. One of the main concerns of a novel molecular diagnostic method is to ensure that the method is optimized and validated with proper and standard controls. In the current study, the nature of samples and/or controls is unclear although the authors declared HBV DNA positive controls and/or positive serum samples in their study. It is uncertain that how the method was set up and optimized. If the method was optimized based on clinical samples, the reference controls used to validate the system are unclear. If the method was optimized based on previously defined reference HBV DNA controls (i.e. plasmids that carry out different HBV genotypes), there is no report on the clinical validation of the assay. Obtaining amplification signals using reference controls would be achieved readily. Thus, there is a flaw in details of assay optimization and validation in the introduced molecular method; specifically, sample and/or reference control collection and preparation, nucleic acid extraction and negative/positive controls were not addressed clearly in this study.

Determination of analytical and clinical sensitivity and specificity, as well as positive and negative predictive value are other important concerns about the molecular diagnostic assay. Although the sensitivity of the method was reported to be 100-200 copies/mL of blood, the calculation method was not mentioned. It would make sense that the authors have used HBV vectors to determine the copy number of each genotype, but it is ambiguous that how this value has been calibrated and then generalized to blood samples. Moreover, there isn’t any report regarding patients’ samples and method of blood HBV DNA extraction. Analytical specificity could be shown by mixture of non-matched HBV DNA genotypes and primers in a same PCR reaction. It is worth noting that most of the similar methods utilize nested PCR approach to increase the sensitivity of the assay for clinical samples. Since the method was not clinically validated based on clinical samples, there is no warranty that the method would be sensitive enough to detect HBV DNA after blood extraction. Consequently, there is no data about positive and negative predictive values based on population study. Once more, this question comes up that if the authors optimized their method based on clinical samples obtained from the Mazandaran University of Medical Sciences, which type of reference controls were employed to validate their method. The authors checked out performance of the assay and showed the accurate intra-run precision of the method by running a triplicate experiment, but this was not tested on clinical samples. Thus, clinical validation of the assay remains to be established as well.

Altogether, when a new molecular diagnostic method is developed, informative methodological references that were used during the optimization and validation process such as analytical sensitivity, specificity, precision, accuracy, and clinical validity should be provided. This would give other researchers confidence in the validity of the techniques, which will encourage future use of such a novel method.
